# Quantitative Structural Analysis of Hyperchromatic Crowded Cell Groups in Cervical Cytology: Overcoming Diagnostic Pitfalls

**DOI:** 10.3390/cancers16244258

**Published:** 2024-12-21

**Authors:** Shinichi Tanaka, Tamami Yamamoto, Norihiro Teramoto

**Affiliations:** 1 Department of Medical Technology, Kawasaki University of Medical Welfare, Kurashiki 701-0193, Japan; 2 Department of Clinical Laboratory, Shikoku Cancer Center, Matsuyama 791-0245, Japan

**Keywords:** hyperchromatic crowded cell groups, texture analysis, cervical cancer

## Abstract

Background: The diagnostic challenges presented by hyperchromatic crowded cell groups (HCGs) in cervical cytology often result in either overdiagnosis or underdiagnosis due to their densely packed, three-dimensional structures. The objective of this study is to characterize the structural differences among HSIL-HCGs, AGC-HCGs, and NILM-HCGs using quantitative texture analysis metrics, with the aim of facilitating the differentiation of benign from malignant cases. Methods: A total of 585 HCGs images were analyzed, with assessments conducted on 8-bit gray-scale value, thickness, skewness, and kurtosis across various groups. Results: HSIL-HCGs are distinctly classified based on 8-bit gray-scale value. Significant statistical differences were observed in all groups, with HSIL-HCGs exhibiting higher cellular density and cluster thickness compared to NILM and AGC groups. In the AGC group, HCGs shows statistically significant differences in 8-bit gray-scale value compared to NILM-HCGs, but the classification performance by 8-bit gray-scale value is not high because the cell density and thickness are almost similar. These variations reflect the characteristic cellular structures unique to each group and substantiate the potential of 8-bit gray-scale value as an objective diagnostic indicator, especially for HSIL-HCGs. Conclusion: Our findings indicate that the integration of gray-scale-based texture analysis has the potential to improve diagnostic accuracy in cervical cytology and break through current diagnostic limitations in the identification of high-risk lesions.

## Simple Summary

In cervical cancer screening, identifying whether certain cell clusters are benign or malignant can be challenging, especially when these clusters are densely packed and appear as “hyperchromatic crowded cell groups”. This study aims to analyze and understand the structural differences in these clusters by using image analysis techniques. By examining factors such as cellular density, cluster thickness, and brightness, the researchers aim to establish a more accurate method for identifying potentially high-risk cells in cervical specimens. This approach could improve diagnostic accuracy, help cytopathologists make more informed decisions and support the development of advanced computerized systems for the early detection of cervical abnormalities. Ultimately, these findings could contribute to earlier, more reliable diagnoses for patients, improving outcomes in cervical cancer care.

## 1. Introduction

The primary objective of cyto-diagnosis is the detection of malignant cells based on their cellular morphological features. The current criteria for the classification of cervical cytology as benign or malignant are limited to the assessment of cellular atypia, including nuclear enlargement, nuclear-to-cytoplasmic ratio, hyperchromasia, and irregular clumpy chromatin [1]. In recent years, liquid-based cytology (LBC), which is known for its high cell collection efficiency, has become the mainstream as a method of choice for preparing cyto-diagnosis specimens, particularly in the cervical region [2,3,4]. With the proliferation of LBC specimens, the frequency of three-dimensional cell clusters appearing on cyto-diagnosis specimens has increased. In conventional cyto-diagnosis where individual cells are observed and cellular atypia is the sole criterion for malignancy determination, it has become challenging to evaluate abnormal densely packed clusters. Those cell clusters are referred to as “hyperchromatic crowded cell groups (HCGs)” on cervical specimens [5]. The presence of HCGs in cervical cytology is associated with a high risk of either overdiagnosis or underdiagnosis, irrespective of whether the underlying condition is benign or malignant. The objective of this study is to quantify the structural organization of HCGs on an individual basis through image analysis and to identify novel cyto-diagnostic indicators for the differentiation of HCGs.

## 2. Overview of Related Work for HCGs

HCGs are widely recognized as a significant factor contributing to the reduced accuracy of cervical cytology [6,7,8]. While numerous reports emphasize the diagnostic challenges posed by HCGs, few studies have delved into their cytomorphological characteristics [6,9,10,11]. Notably, no studies have specifically analyzed the structural organization of HCGs. Previous investigations have primarily focused on HCGs in high-grade squamous intraepithelial lesion (HSIL) or cases classified as negative for intraepithelial lesion or malignancy (NILM), with little attention to HCGs in glandular tumors. Moreover, while cytologic analyses have been conducted, no substantial proposals have been made to enhance diagnostic accuracy [11].

In recent years, there has been growing interest in the application of deep learning for cytological diagnosis across various cancers [12,13,14,15]. These studies demonstrate high diagnostic performance; however, it has been shown that even deep learning models trained on extensive datasets tend to misclassify cells within clusters. Such misclassifications can occur in both squamous and glandular cell types [12]. This limitation underscores the need for further refinement in diagnostic approaches.

Additionally, image analysis in cytological diagnosis has traditionally been limited to cellular findings obtained from individual cells, such as nuclear size, nuclear shape, and chromatin distribution [16,17]. Our study addresses the limitations of prior research by analyzing the structural characteristics of HCGs, focusing on their potential implications for improving diagnostic accuracy.

## 3. Materials and Methods

### 3.1. Subjects

The present study was performed in accordance with the Declaration of Helsinki. It was approved by the Institutional Ethics Committee of Shikoku Cancer Center (Ehime, Japan; No.2024-503) and by the Institutional Ethics Committee of Kawasaki University of Medical Welfare (Okayama, Japan; No. 24-011). A retrospective study design was used.

Data comprising a total of 585 HCGs images from 71 cervical cytology samples were retrospectively collected from the laboratory of Shikoku Cancer Center. The study included 175 image data samples from 28 cases of NILM, 202 image data samples from 19 cases of atypical glandular cell (AGC), and 208 image data samples from 24 cases of HSIL. In this study, adenocarcinoma in situ and adenocarcinoma were collectively defined as AGC. All women undergoing colposcopy were included in the analysis. Patients with tumor lesions, including HSIL and AGC, were identified by biopsy or surgical specimens within three months after the cyto-diagnosis. The study consisted of 28 cases of NILM, defined as the absence of tumor lesions by colposcopy and iterative cytology over a one-year period. The origins of NILM-HCGs may include metaplastic cells, atrophic epithelium, endometrial stroma, and cervical glandular epithelium. In this study, NILM-HCGs were limited to morphologically classifiable clusters, and the focus was placed on immature or mature metaplastic cell clusters (Metaplastic) and benign glandular cell clusters (Glandular), which are often challenging to differentiate as benign or malignant in cytological diagnosis.

### 3.2. Specimen Preparation and Staining

Cervical samples were collected using a Cervex-Brush^®^ and preserved in a BD SurePath^TM^ vial. To detach the cells that had adhered to the brush, the sample was vortexed using a vortex mixer. A uniform cell suspension was prepared with BD PrepMate™ (Nippon Becton Dickinson Company, Ltd., Tokyo, Japan), and artifacts such as blood components and mucus were removed. The cells were evenly smeared using a BD PrepStain™ system (Nippon Becton Dickinson Company, Ltd., Tokyo, Japan). Those steps were conducted in accordance with modified manual protocols. Nuclear staining was performed using Gill’s Hematoxylin V (Muto Pure Chemicals, Ltd., Tokyo, Japan), while cytoplasmic staining was conducted with OG-6 (Muto Pure Chemicals, Ltd., Tokyo, Japan) and EA-50 (Muto Pure Chemicals, Ltd., Tokyo, Japan).

### 3.3. Texture Image Analysis

High-resolution digital images of HCGs were captured using an OLYMPUS DP27 microscope camera and the cellSens Dimension (Olympus Corporation, Tokyo, Japan) imaging system with a 20× objective lens, resulting in images with a resolution of 2448 × 1920 pixels. The imaging conditions were as follows: (1) Exposure time: 1.961 ms. (2) Sensitivity: the ISO setting was 100. (3) High dynamic range: disabled. (4) Light source: fixed. (5) Numerical aperture: 0.50. The acquired HCGs images were analyzed using ImageJ 1.54, an image processing and analysis software (JAVA) developed by the National Institutes of Health. The analysis involved manually trimming the target areas using the freehand selection and measurement tools in ImageJ, followed by various texture image analyses.

#### 3.3.1. Eight-Bit Gray-Scale Value

The acquired 24-bit images were converted to 8-bit images using ImageJ software. HCGs, the subject of analysis, were manually trimmed using a Wacom intuos Pro as a pen tablet. The numerical distribution of HCGs represented in 8-bit color was analyzed, and the mode of this distribution was defined as the 8-bit gray-scale value. The conversion of cytological images to 8-bit format has been applied as an indicator of nuclear chromatin density [16,17]. In this study, we applied this technique to three-dimensional clusters to quantify their structural organization.

#### 3.3.2. Thickness

To analyze the thickness of the samples, the HCGs were subdivided into 40 layers along the *Z*-axis, at 1 μm intervals using a virtual slide scanner (Aperio AT2, Leica Biosystems, Buffalo Grove, IL, USA). The distance from the deepest layer, where the cells at the bottom of the slide are in focus, to the topmost layer, where the uppermost portion of the cluster is in focus, was measured.

#### 3.3.3. Skewness

The skewness value was calculated using Microsoft Excel software (Microsoft Corporation, Redmond, WA, USA). The function “SKEW” was applied to the dataset.

#### 3.3.4. Kurtosis

The kurtosis value was calculated using Microsoft Excel software (Microsoft Corporation, Redmond, WA, USA). The function “KURT” was applied to the dataset.

### 3.4. Statistical Analysis

Data analysis was conducted using R software (version 4.3.2, R Foundation for Statistical Computing, Vienna, Austria). Based on the distribution characteristics of the data, the Kruskal–Wallis test was employed to assess differences among the groups for data that did not follow a normal distribution. Subsequent post hoc analysis using the Steel–Dwass test demonstrated that the groups exhibited statistically significant differences. Furthermore, correlation analysis of thickness, skewness, and kurtosis was conducted, and the effects of these variables on the 8-bit gray-scale value were evaluated using multiple regression analysis. A *p*-value of less than 0.05 was considered statistically significant.

## 4. Results

**Figure 1:**
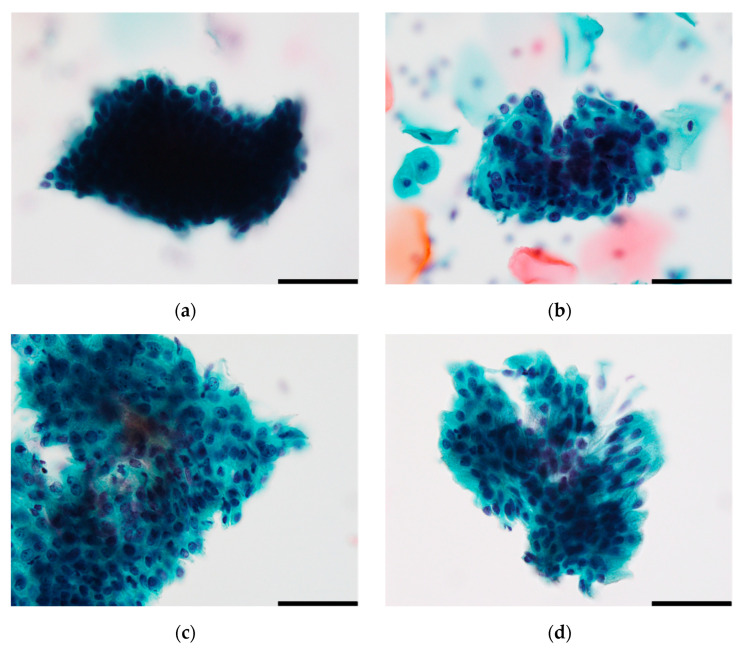
Representative 24-bit images of HCGs. (**a**) HSIL-HCGs; (**b**) AGC-HCGs; (**c**) NILM-HCGs for immature metaplastic cells; (**d**) NILM-HCGs for benign glandular cells. Scale bar: 50 µm.

Representative images of HCGs are shown in Figure 1; (a) depicts HSIL, (b) shows AGC, (c) presents immature metaplastic cells, and (d) represents benign glandular cells. In all the images, the focus is not on the scattered background cells, with the objective of highlighting the three-dimensional structure.

The analysis of the 8-bit gray-scale distribution is shown in Table 1. The Kruskal–Wallis test indicated *p*-values below 0.01, demonstrating statistically significant variations among the HSIL-HCGs, AGC-HCGs, NILM-HCGs, Metaplastic, and Glandular groups. It is noteworthy that the HSIL-HCGs group exhibited the lowest 8-bit gray-scale value, with a median of 16 and an interquartile range (IQR) of 10–25. Moreover, the results of post hoc analysis indicate that AGC-HCGs show significant differences compared to most groups, including Glandular, HSIL-HCGs, Metaplastic, and NILM-HCGs. Additionally, HSIL-HCGs exhibits significant differences with the majority of groups, particularly displaying large mean differences when compared to Metaplastic, NILM-HCGs, and AGC-HCGs. Conversely, no significant differences are observed between Glandular and NILM-HCGs or between Metaplastic and NILM-HCGs, suggesting that these groups may exhibit similar characteristics.

**Figure 2:**
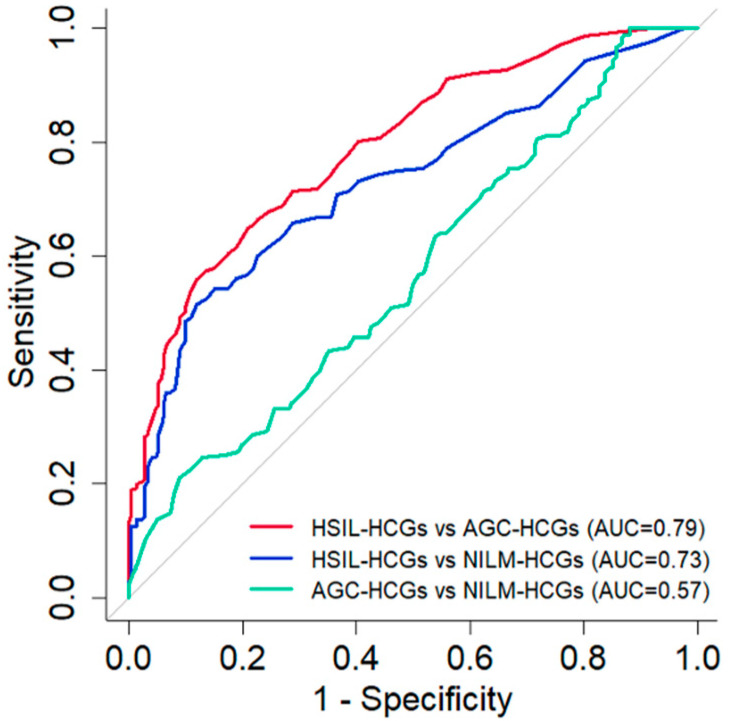
Receiver operating characteristic curves for the pairwise classification performance between HSIL-HCGs, AGC-HCGs, and NILM-HCGs based on the 8-bit gray-scale value. The red line represents the ROC curve for HSIL-HCGs vs. AGC-HCGs (AUC = 0.79), the blue line represents HSIL-HCGs vs. NILM-HCGs (AUC = 0.73), and the green line represents AGC-HCGs vs. NILM-HCGs (AUC = 0.57). The diagonal gray line represents the random classification threshold (AUC = 0.5).

From Table 1 and Table 2, no significant differences in the measured values by NILM-HCGs’ origin were observed. Therefore, in the exploratory analysis of the cyto-diagnostic significance and influencing factors of the 8-bit gray-scale value, NILM-HCGs were treated as a single unified group without further subdivision. Receiver operating characteristic (ROC) curves were generated to evaluate the classification performance between HSIL-HCGs, AGC-HCGs, and NILM-HCGs (Figure 2), with HSIL-HCGs vs. AGC-HCGs showing an AUC of 0.79, sensitivity of 0.56, and specificity of 0.88 at a cut-off value of 33.5. Similarly, HSIL-HCGs vs. NILM-HCGs showed an AUC of 0.73, sensitivity of 0.51, and specificity of 0.88 at the same cut-off value of 33.5, and AGC-HCGs vs. NILM-HCGs showed an AUC of 0.57, sensitivity of 0.21, and specificity of 0.91 at a cut-off value of 13.5. These results indicate a relatively high discriminatory power between HSIL-HCGs and AGC-HCGs, as well as HSIL-HCGs and NILM-HCGs, compared to the weaker classification performance between AGC-HCGs and NILM-HCGs.

**Figure 3:**
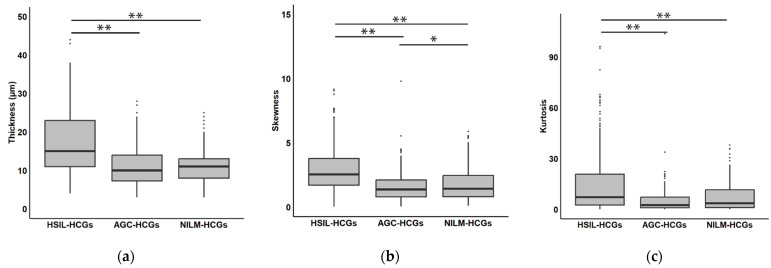
A comparative analysis of the thickness, skewness, and kurtosis of each group. The box plots illustrate the distribution of (**a**) thickness, (**b**) skewness, and (**c**) kurtosis for HCGs. The boxes show the interquartile range (IQR), with the whiskers extending to the minimum and maximum values. The median values are indicated by the lines inside the boxes, facilitating a comprehensive comparison of the texture analysis parameters across the samples. * *p* < 0.05, ** *p* < 0.01, Steel–Dwass test.

**Figure 4:**
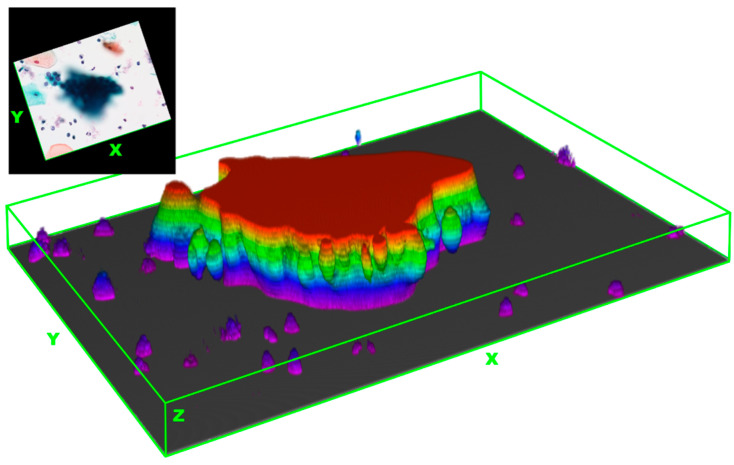
Three-dimensional stack image of HSIL-HCGs. The presented figure is a Z-stack image of HSIL-HCGs, with a total thickness of 29 µm. The image was created by capturing still images at 1 µm intervals and converting them into a composite stack using ImageJ. Pseudocolor was applied to each layer, with a color gradient representing the thickness, thereby providing a clear visualization of the three-dimensional structure of the HSIL-HCGs. The accompanying inset shows a two-dimensional image of the same HSIL-HCGs for comparison.

As illustrated in Table 3, the results indicate that the median thickness of HSIL-HCGs was 15 µm (IQR: 11–23 µm), which was higher compared to AGC-HCGs at 10 µm (IQR: 8–14 µm) and NILM-HCGs at 11 µm (IQR: 8–13 µm). The HSIL-HCGs exhibited statistically significant differences in comparison to the other two groups (Figure 3. *p* < 0.01, Steel–Dwass test). With regard to thickness, as shown in Figure 4, HSIL-HCGs are distinctly three-dimensional clusters.

**Figure 5:**
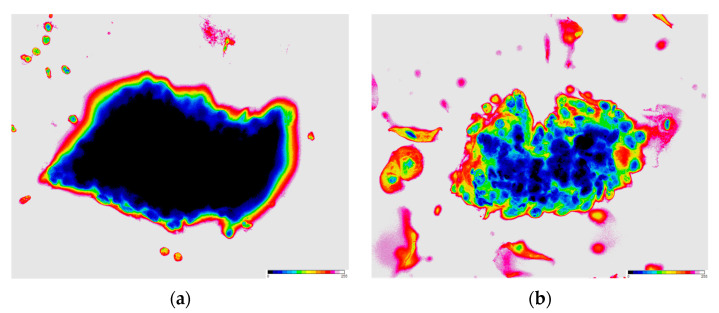
A 16-color gradient applied to 8-bit images of HSIL-HCGs and AGC-HCGs. (**a**) HSIL-HCGs; (**b**) AGC-HCGs. The color gradient enhances the visualization of the differences in gray-scale intensity, providing a clearer distinction between the two types of HCGs. The 16-color scheme helps to highlight structural and textural variations in the cells. The nuclear regions display relatively darker colors, whereas the cytoplasmic regions exhibit colors shifted to the right of the scale bar’s center.

**Figure 6:**
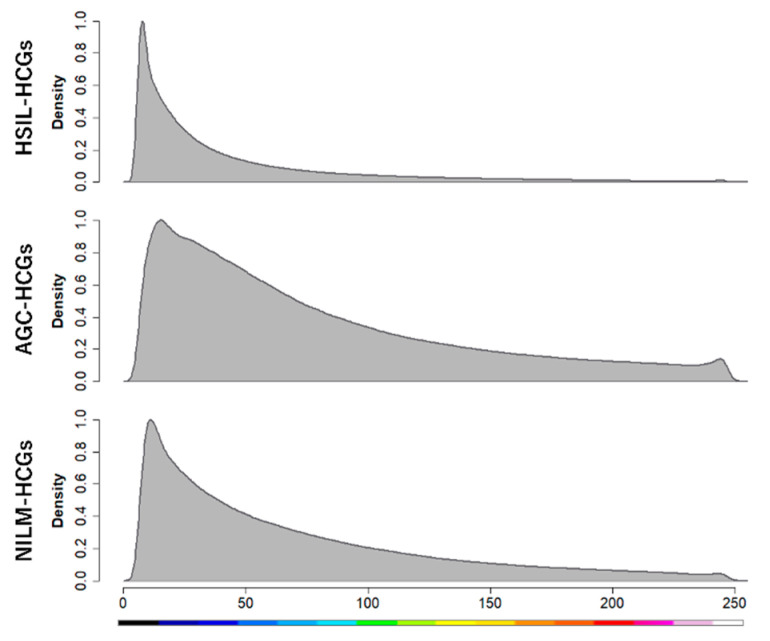
Density distribution of measurements across three groups. The histogram was generated by combining the measurement values from each group, providing an overview of the distribution patterns.

Furthermore, as indicators of cell density, skewness and kurtosis were calculated. The skewness of the 8-bit gray-scale histogram exhibited variability across the groups, with a value of 2.55 (IQR: 1.72–3.84) for HSIL-HCGs, 1.28 (IQR: 0.59–1.92) for AGC-HCGs, and 1.37 (IQR: 0.76–2.40) for NILM-HCGs. The kurtosis values also showed considerable differences, with HSIL-HCGs significantly higher at 6.12 (IQR: 2.06–16.89), AGC-HCGs at 0.61 (IQR: −0.72–3.07), and NILM-HCGs at 0.64 (IQR: −0.65–5.37). Statistical analysis revealed that HSIL-HCGs were significantly darker and more concentrated cell clusters (Figure 3. *p* < 0.01, Steel–Dwass test). Figure 5 depicts representative HSIL-HCGs and AGC-HCGs colored with 256-level pseudocolor. Additionally, a combined histogram is presented in Figure 6. The probability distribution in AGC-HCGs is observed, and a higher density is evident in the range of 50 to 250 levels compared to the other groups.

**Figure 7:**
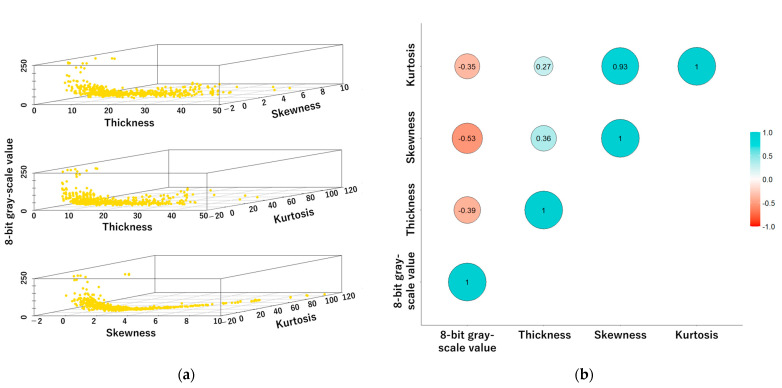
Correlation and multiple regression analysis of factors influencing 8-bit gray-scale value. (**a**) A three-dimensional scatter plot showing relationships between each factor and 8-bit gray-scale value. (**b**) Correlation coefficient visualization using bubble plot. The analysis elucidates the significant relationships between the variables, thereby providing insights into the manner in which thickness, skewness, and kurtosis contribute to the 8-bit gray-scale value. The regression model quantifies the impact of these factors on the 8-bit gray-scale value.

Finally, Figure 7 presents the results of the correlation analysis between thickness, skewness, and kurtosis. Thickness, skewness, and kurtosis exhibited negative correlations with the 8-bit gray-scale value. A strong positive correlation (r = 0.93) was observed between skewness and kurtosis. Additionally, the data demonstrate significant relationships between these variables, with multiple regression analysis yielding a multiple R-squared value of 0.55. Thickness, skewness, and kurtosis were all found to be significant predictors of 8-bit gray-scale value (Table 4. *p* < 0.01 for each variable).

## 5. Discussion

This study employed a comprehensive image analysis approach to investigate the structural organization of HCGs. The image analysis methods used in this study, including 8-bit gray-scale value, thickness, skewness, and kurtosis, revealed clear structural differences in HCGs in each group (NILM, AGC, HSIL). In HSIL-HCGs, all items showed marked differences from the other groups. In particular, the 8-bit gray-scale value is of significant diagnostic value in distinguishing. The markedly lower 8-bit gray-scale value observed in HSIL-HCGs (median 16, IQR: 10–25) suggests that this metric may serve as a reliable indicator of cervical squamous epithelial tumor. Similarly, the higher 8-bit gray-scale value observed in AGC-HCGs (median 36.5, IQR: 21–60) indicates its potential as an indicator for cervical glandular tumors (Table 1 and Table 2). Furthermore, as shown in Figure 2, 8-bit gray-scale value has high diagnostic value for HSIL-HCGs. Therefore, 8-bit gray-scale analysis for HCCs can serve as a valuable diagnostic tool, offering an objective measure for cytological evaluation. Additionally, texture image analysis of HCGs revealed factors associated with the 8-bit gray-scale value.

It is postulated that the thickness and cellular density of HCGs exert a substantial influence on the determination of the 8-bit gray-scale value. It is presumed that the augmented thickness is attributable to the three-dimensional clustering that is characteristic of dysplastic cells. This increase in cellular aggregation is reflected in darker gray-scale values in cytological images, thereby facilitating clearer differentiation. At present, there is no established method for quantifying cellular density in three-dimensional cell clusters within cytological specimens. In the present study, we calculated skewness and kurtosis from histograms generated from 8-bit images of HCGs. Skewness represents the asymmetry of the histogram, with positive values indicating a leftward skew, which, in this study, corresponds to an 8-bit gray-scale value close to “0”. Kurtosis indicates the sharpness of the histogram, with higher values representing greater sharpness. Consequently, HCGs with high skewness and kurtosis values are identified as dark clusters. As illustrated in Figure 5, these dark areas correspond to regions with a higher number of cell nuclei. This suggests that skewness and kurtosis can serve as indicators of cellular density.

Upon further analysis of the results, the HSIL-HCGs exhibited higher thickness values and were thicker than the other groups (Table 3 and Figure 3). Moreover, the skewness and kurtosis measurements exhibited high values. This suggests the presence of a markedly elevated cellular density. Squamous epithelium is an epithelium that is resistant to physical stimuli due to its stratification, which eliminates the presence of intercellular spaces. The HSIL cells are characterized by a high nucleus-to-cytoplasm ratio and a paucity of cytoplasmic regions [18,19]. It is therefore evident that when atypical cells with these morphological characteristics manifest as clusters, they tend to form thicker and more cell dense clusters. The findings of this study align with the aforementioned theory. In contrast, NILM-HCGs and AGC-HCGs exhibited a tendency towards reduced thickness and cellular density. The non-tumorigenic cases, designated as NILM-HCGs, demonstrated a lack of excessive proliferation, resulting in thinner clusters with lower cellular density. Despite sharing tumor-forming factors with HSIL-HCGs, AGC-HCGs exhibited statistically significant differences across all parameters. We hypothesize that these differences are largely influenced by the distinct roles and functions of the epithelial cell types involved. As previously stated, squamous epithelial cells are inclined to form high-density, three-dimensional cell clusters. On the other hand, glandular cells, which are responsible for secreting mucus and other substances, are less prone to stratification. Furthermore, glandular cells maintain cytoplasmic regions essential for protein synthesis and form ducts to expel secretions [20]. This functional feature persists in cervical glandular tumors [21,22]. Therefore, despite exhibiting irreversible overproliferation, AGC-HCGs are relatively thin and exhibit lower cellular density compared to HSIL-HCGs.

In regard to the diagnostic significance of structural image analysis for HCGs, the accuracy of conventional cervical cyto-screening for the detection of HSIL lesions and AGC lesions is demonstrably inadequate [23,24]. One of the factors that reduces the accuracy of cervical cytology is HCGs [25,26]. While p16/Ki67 and PHH3 immunocytochemical staining are recommended for challenging cyto-diagnostic cases, their practicality may be limited due to the high costs associated with specialized equipment and reagents [27,28,29,30]. Evered A et al. [11] reported the results of morphological analysis of HCGs. The researchers assessed variables such as size, shape, and color intensity of HCGs to determine their utility in distinguishing between different cytological categories. Although nine out of ten variables were found to contribute significantly to the discriminant model, no single factor was identified as having sufficient power for reliable classification. In this study, based on the 8-bit gray-scale value of NILM-HCGs, HSIL-HCGs demonstrated lower values, allowing for clear differentiation. This distinction indicates the potential for effectively distinguishing between benign and malignant lesions, especially squamous epithelial tumors. Previous studies have emphasized that 8-bit gray-scale analysis at the cellular level can be used to identify chromatin condensation and increased chromatin density as indicators of malignancy, underscoring the significance of 8-bit gray-scale analysis in cytology [16]. The 8-bit gray-scale value provides a quantitative proxy for chromatin condensation, thickness, and cellular density, all of which are critical features in cyto-diagnosis. To the best of our knowledge, there are no previous reports that have applied the 8-bit gray-scale value for the purpose of quantifying architectural features. Consequently, our study provides highly novel research findings. Furthermore, in this study, thickness, skewness, and kurtosis were identified as significant factors influencing the 8-bit gray-scale value, thereby further substantiating its diagnostic utility. As shown in Figure 7, the results of the multiple regression analysis indicated that skewness was the most significant factor influencing the 8-bit gray-scale value. Moreover, a strong positive correlation was observed between skewness and kurtosis (r = 0.93). These findings suggest that cellular density exerts an influence on the 8-bit gray-scale value. With regard to thickness, HCGs with high values tend to exhibit low 8-bit gray-scale values. However, at lower thickness values (10 µm or less), there is notable variability (Figure 7). This further emphasizes that cellular density plays a significant role in influencing the 8-bit gray-scale value. In this study, we focused on the 8-bit gray-scale value, with AGC-HCGs showing significantly higher values than the other groups (Table 2); however, the classification performance for NILM-HCGs was low (Figure 2). Therefore, as shown in Figure 6, we focused on the distinctive histograms of AGC-HCGs exhibiting the following characteristics: (1) a higher density proportion in the cytoplasmic region with 8-bit gray-scale value between 100 and 200 and (2) a gradual slope from the peak value. In AGC-HCGs, it is expected that not only the 8-bit gray-scale value but also these characteristic features serve as valuable diagnostic criteria for distinguishing AGC-HCGs.

In recent years, research on the use of AI for automated differentiation in cervical cytology has become a prominent area of investigation, as evidenced by the growing number of publications in this field [12,31,32,33]. Nevertheless, as with human microscopic assessment, the accuracy of HCGs identification remains a limiting factor in diagnostic precision for AI image recognition [12]. The results of structural analysis of HCGs across different groups obtained in this study are anticipated to facilitate enhancements in AI image recognition. The accuracy of AI image recognition is contingent upon supervised learning. In the absence of a clear understanding of the features to be utilized for training, the accuracy of AI image recognition is diminished [34]. The data obtained from our texture image analysis are likely to represent a key feature for AI training. One promising approach is to integrate texture features into pre-trained models such as ResNet, VGG, and EfficientNet. By incorporating these handcrafted features into the output or middle layer of a deep learning architecture, the feature space of the model can be enriched with domain-specific information. Transfer learning allows these models to be adapted to datasets with unique texture patterns, which may improve classification performance, especially for complex cyto-diagnostic images. The ability of the 8-bit gray-scale value to consistently distinguish between NILM-HCGs and HSIL-HCGs bears considerable clinical significance. When combined with thickness, skewness, and kurtosis, this metric provides an objective and reproducible diagnostic framework. The results of this study, which concentrate on HCGs that were challenging to distinguish morphologically, may prove valuable in establishing new diagnostic criteria in clinical practice. The integration of these quantitative parameters into routine cytology workflows is anticipated to reduce diagnostic variability and improve the accuracy of high-risk lesion detection. Furthermore, this approach could contribute to the construction of high-performance deep learning models in the future.

A limitation of our study is that the multiple R-squared value of the regression model in this study was 0.55, indicating a moderate level of fit. This suggests that additional factors may influence the dependent variable beyond the variables of thickness, skewness, and kurtosis. Further examination, such as exploring additional explanatory variables and non-linear relationships among variables, is warranted to enhance the model’s predictive capability. Caution should be exercised when interpreting the model’s results due to the moderate level of explained variance. Further investigation of these factors is necessary in future studies. Furthermore, a future challenge is the application of deep learning for automatic identification. For this purpose, higher-dimensional texture image analysis such as a gray level co-occurrence matrix and local binary patterns will be necessary.

## 6. Conclusions

HSIL-HCGs exhibited thicker, more dense profiles (higher skewness and kurtosis), while AGC-HCGs demonstrated similar thicknesses but lower cellular density profiles (lower skewness) compared to NILM-HCGs. These characteristics can be highlighted using the 8-bit gray-scale value. It is our contention that this metric offers a practical solution to some of the pitfalls in cervical cytology.

## Figures and Tables

**Table 1:** Eight-bit gray-scale distribution among HSIL-HCGs, AGC-HCGs, and NILM-HCGs.

	8-bit Gray-Scale Value		Kruskal–Wallis Test, *p* Value
	Median Value	IQR ^1^	
HSIL-HCGs	16	10–25	<0.01
AGC-HCGs	36.5	21–60	
NILM-HCGs	34	17–51	
Metaplastic	34	18–53	
Glandular	32	15–45	

NILM-HCGs were further divided into two groups based on their origin: the Metaplastic group, consisting of immature metaplastic and metaplastic cells, and the Glandular group, consisting of benign glandular epithelial cells. ^1^ IQR: interquartile range.

**Table 2:** Comparison of 8-bit gray-scale value of HSIL-HCGs, AGC-HCGs, and NILM-HCGs using Steel–Dwass test.

Steel–Dwass Test			
Variable 1	Variable 2	*z* Value	*p* Value
AGC-HCGs	Glandular	1.82	0.03
AGC-HCGs	HSIL-HCGs	10.45	<0.01
AGC-HCGs	Metaplastic	1.93	0.03
AGC-HCGs	NILM-HCGs	2.30	0.01
Glandular	HSIL-HCGs	3.88	<0.01
Glandular	Metaplastic	−0.62	0.24
Glandular	NILM-HCGs	−0.50	0.31
HSIL-HCGs	Metaplastic	−7.46	<0.01
HSIL-HCGs	NILM-HCGs	−7.74	<0.01
Metaplastic	NILM-HCGs	0.21	0.42

**Table 3:** The results of texture image analysis for HSIL-HCGs, AGC-HCGs, and NILM-HCGs.

	Thickness, Median Value (IQR)	Skewness, Median Value (IQR)	Kurtosis, Median Value (IQR)
HSIL-HCGs	15 µm (11–23 µm)	2.55 (1.72–3.84)	6.12 (2.06–16.89)
AGC-HCGs	10 µm (8–14 µm)	1.28 (0.59–1.92)	0.61 (−0.72–3.07)
NILM-HCGs	11 µm (8–13 µm)	1.37 (0.76–2.40)	0.64 (−0.65–5.37)

**Table 4:** Multiple regression analysis for 8-bit gray-scale value using thickness, skewness, and kurtosis.

Variable	Estimate	Std. Error	*t* Value	*p* Value
Intercept	140.29	5.611	25.00	<0.01
Thickness	−4.09	0.49	−8.27	<0.01
Skewness	−65.24	4.04	−16.16	<0.01
Kurtosis	8.47	0.80	10.64	<0.01
Thickness–Skewness	1.93	0.29	6.64	<0.01
Thickness–Kurtosis	−0.18	0.03	−5.68	<0.01
Skewness–Kurtosis	−0.33	0.06	−5.21	<0.01

Multiple R-squared is 0.55. The regression model incorporates both the main effect terms; thickness, skewness, kurtosis, and the interaction terms; thickness–skewness, thickness–kurtosis, and skewness–kurtosis.

## Data Availability

All data generated or analyzed during this study are included in this article. Further inquiries can be directed to the corresponding author.
